# Improving the anti-ageing activity of coenzyme Q10 through protransfersome-loaded emulgel

**DOI:** 10.1038/s41598-021-04708-4

**Published:** 2022-01-18

**Authors:** Qurrota Ayunin, Andang Miatmoko, Widji Soeratri, Tristiana Erawati, Joni Susanto, Djoko Legowo

**Affiliations:** 1 Master Program of Pharmaceutical Sciences, Department of Pharmaceutical Sciences, Faculty of Pharmacy, Universitas Airlangga, Nanizar Zaman Joenoes Building, Campus C Mulyorejo, Surabaya, 60115 Indonesia; 2 Faculty of Pharmacy, Hospital Administration, Public Health, and Radiology, Study Program of Pharmacy, Institut Ilmu Kesehatan STRADA, Jl. Manila 37, Kediri, 64133 Indonesia; 3 Department of Pharmaceutical Sciences, Faculty of Pharmacy, Universitas Airlangga, Nanizar Zaman Joenoes Building, Campus C Mulyorejo, Surabaya, 60115 Indonesia; 4 Department of Anatomy and Histology, Faculty of Medicine, Universitas Airlangga, Jl. Mayjen. Prof. Dr. Moestopo No. 47, Campus A Mulyorejo, Surabaya, 60132 Indonesia; 5 Department of Veterinary Pathology, Faculty of Veterinary Medicine, Universitas Airlangga, Jl. Mayjen. Prof. Dr. Moestopo No. 47, Campus C Mulyorejo, Surabaya, 60115 Indonesia

**Keywords:** Diseases, Medical research, Nanoscience and technology

## Abstract

Coenzyme Q10 (CoQ10) is a naturally produced organic molecule which acts as an antioxidant agent, including in skin anti-ageing, and plays a major role in the social determinants of health. However, its level in the body will decrease during ageing. Therefore, an external supplement is required to repair damaged skin, especially the skin dermis layer. This study aims to evaluate the use of a protransfersomal emulgel to improve the skin delivery and stability of CoQ10 which demonstrates low water solubility, poor permeability and instability. CoQ10 was initially dissolved in oleic acid at a weight ratio of 1:56. Protransfersome was then loaded with CoQ10 (Protransf-CoQ10) and prepared using a composition of L-α-Phosphatidylcholine and Tween 80 at a molar ratio of 85:15. The Protransf-CoQ10 was dispersed in an emulgel base consisting of Tween 80 and Span 80 to produce Protransf-CoQ10 emulgel. The in vivo studies of anti-aging activity and irritability were further evaluated by applying daily 200 mg of emulgels twice a day to a 4 cm^2^ section on the back of a UV-ray aging-induced male Balb/c mouse 20 min before irradiation. The results showed that Protransf-CoQ10 could transform into transfersomal vesicles with particle sizes of approximately 201.5 ± 6.1 nm and a zeta potential of − 11.26 ± 5.14 mV. The dispersion of Protransf-CoQ10 into emulgel base resulted in stable Protransf-CoQ10 Emulgel during 28 days of observation at low temperatures. Moreover, the in vivo study revealed that Protransf-CoQ10 Emulgel successfully increases the collagen density and number of fibroblast cells in UV radiation skin-aged induced-mice which reflects its potential for repairing the skin ageing process. In addition, the 24-h topical application of Protransf-CoQ10 Emulgel showed that no erythema or skin rash was observed during the study. In conclusion, loading CoQ10 into protransfersomal Emulgel successfully enhanced the stability and anti-ageing efficacy enabling its potential use as anti-ageing cosmetics.

## Introduction

Premature skin ageing occurs because the skin, as the outermost organ, is always directly exposured to oxidants in the environment and is frequently a determining factor in social life. In addition, with increasing age, the activity of mitochondria in the body as a producer of energy in regenerating cells and tissues decreases^1^. Both these internal and external factors cause impaired tissue function and structural changes^2^ culminating in skin ageing characterized by thinning of the epidermis and skin dermis and, ultimately, resulting in wrinkles, fine facial lines, and loss of elasticity^3,4^. Skin elasticity is largely dependent upon young collagen fibers and fibroblasts, collagen-producing cells in the dermis layer, whose numbers decrease during the ageing process^5^.

Anti-ageing cosmetics have been widely used to promote skin regeneration, especially of the upper skin layers which protect the skin against dehydration, penetration by various microorganisms, allergens, irritants, reactive oxygen species (ROS) and radiation, thereby maintaining healthy skin^6^. Coenzyme Q10 (CoQ10) is one of the natural compounds often employed as an antioxidant, which plays a key role in stabilizing plasma and other intracellular membranes that protect against membrane phospholipid peroxidation^7^. CoQ10 acts by maintaining skin quality against free radicals^3^ which have been known to activate the mitogen-activated protein kinase (MAPK) pathway that produces matrix metalloproteinases (MMPs) such as collagenase, thus damaging collagen fibers^8–10^. During ageing, the levels of CoQ10 in organs, including the skin, also decrease with the result that it is necessary to supply CoQ10 to achieve normal levels of between 0.50 and 1.65 μg/mL within the body. Topical administration of CoQ10 has been shown to be effective in reducing wrinkles in skin that has been exposed to UV rays^3^.

CoQ10 demonstrates low solubility in water (0.193 μg/mL) with a large molecular weight of 863.36 g/mol and high lipophilicity with a log P value of 21. This limits its penetration of the skin and explains it tendancy to be deposited in the stratum corneum^11,12^. Moreover, CoQ10 decomposes when exposed to light^13^. Loading CoQ10 into protransfersome, a vesicular carrier would probably constitute an effective strategy to enhance its biological activity within the skin in addition to increasing its stability.

Protransfersome, one of the provesicular nanocarriers that provides superior skin penetration and high stability, is widely used in transdermal delivery^14^. It possesses a flattened liquid crystal structure which is converted into an ultraflexible vesicle known as transfersome through the absorption of water from the skin during in situ hydration^15–17^. Transfersome is known to be an ultradeformable vesicle which is highly flexible and deformable, rendering it capable of passing through three skin penetration pathways^18^. Transfersome can rapidly penetrate the stratum corneum and enter the deeper skin layers via the intercellular lipid of the stratum corneum. It can fuse with the cell membrane, enabling it to enter the transcellular pathway, and is able to penetrate intact through the hair follicle pathway to penetrate the deeper layers of the skin^19–21^. Protransfersome is composed of amphiphatic lipid components such as phosphatidylcholine which, significantly, form double-layer membrane of vesicles, and surfactant as an edge activator that increases the vesicle flexibility or deformability^22^. In general, protransfersome contains a larger number of phospholipids than that present in transfersomes. During the manufacturing process, the protransfersome does not undergo an extrusion process to produce unilamellar vesicles as observed in the transfersome. This is because the protransfersome is a provesicular carrier system which will be converted into transfersome after it comes into contact with water in situ^23^. Therefore, under a light microscope, the protransfersome can be seen to possess a palisade crystalline liquid form, whereas transfersomes are vesicular when in liquid media^24^.

The use of ultradeformable vesicles has successfully improved the skin penetration of drugs and efficacy of anti-ageing properties of certain antioxidant molecules such as tocopherol which, when prepared in transfersome, possess good characteristics with a particle size < 100 nm and entrapment efficiency of up to 90%. Moreover, it is well distributed within the skin layer and in vitro tests have proved it biocompatible with keratinocytes and fibroblasts, indicating its protective effect against oxidative damage and the potential for wound healing^25^. Previous reports have evaluated the use of nanocarriers for CoQ10 delivery such as a self-emulsifying drug delivery system (SEDDS)^26^, ethosomes^27^, transethosomes^28^, and microemulsion^29^. The use of transethosomes successfully encapsulated CoQ10 up to 97% in vesicles and produced > 95% drug deposition in different skin layers resulting in high efficacy for androgenic alopecia^28^. The low water solubility of CoQ10 frequently limits drug encapsulation efficiency in nanocarriers, thus the use of large amounts of lipid phase or ethanol may improve its loading.

In this study, a protransfersome containing CoQ10 will be prepared for anti-ageing emulgel. The high level of phospholipids contained in protransfersome is intended to improve drug loading. The use of protransfersome in the anti-ageing activity and irritation level of Protransf-CoQ10 emulgel was evaluated in vivo using UV-induced aged mice models. This study could represent an attempt to improve CoQ10 anti-ageing activity with the result that is effective, safe and non-irritating.

## Results

This study aims to evaluate the potential use of protransfersome for topical delivery of CoQ10 as an anti-ageing agent. This study provides a scientific approach to successfully delivering low water solubility and poor permeable lipophilic substances and nanovesicular carriers specifically designed for anti-ageing cosmetics. The CoQ10 was loaded into protransfersomal emulgel composed of oleic acid containing soluble CoQ10, phospholipids as bilayer-forming lipids, and Tween 80 which acts as the edge activator of bilayer membrane after the protransfersome has been hydrated with skin water in situ, before being loaded into an emulgel base*.* There were improvements in stability and potential efficacy to inhibit premature ageing of the skin in UV-radiation skin aged-induced mice models as demonstrated in this study.

### Physical characteristics and stability of protransfersome-loaded CoQ10 emulgel

**Figure 1:**
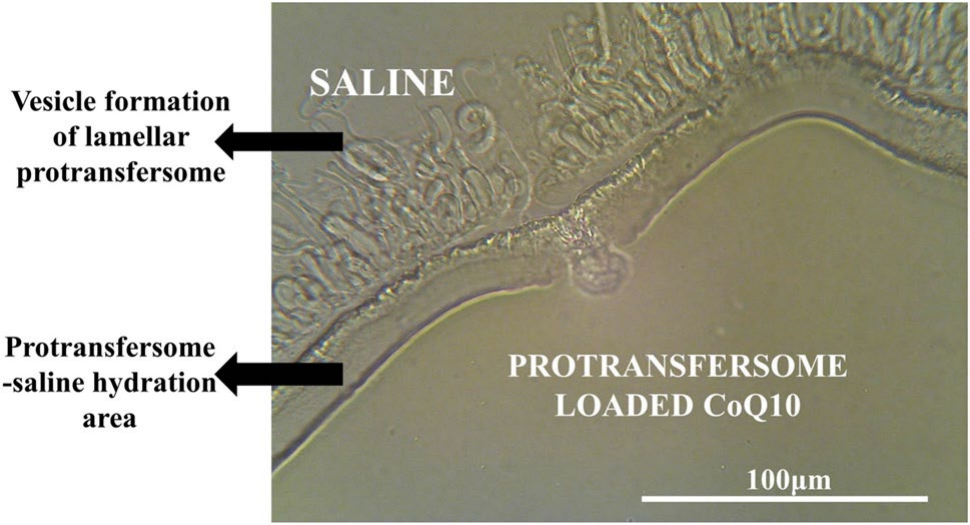
Lamellar structure of liquid crystals of the CoQ10 protransfersome emulgel after adding one drop of saline under an optical microscopy observation at × 400 magnification (Scale bar:100 μm).

After dissolving the CoQ10 in oleic acid and encapsulated it into protransfersomes composed of phospholipids and Tween 80, the protransfersome-loaded CoQ10 (Protransf-CoQ10) forms a bright orange, viscous, oily liquid, with a distinctive phospholipid smell, and viscous consistency. After hydration with saline, lamellar vesicular structures rapidly formed and were ultimately transformed into transfersome vesicles, as shown in Fig. 1.

**Figure 2:**
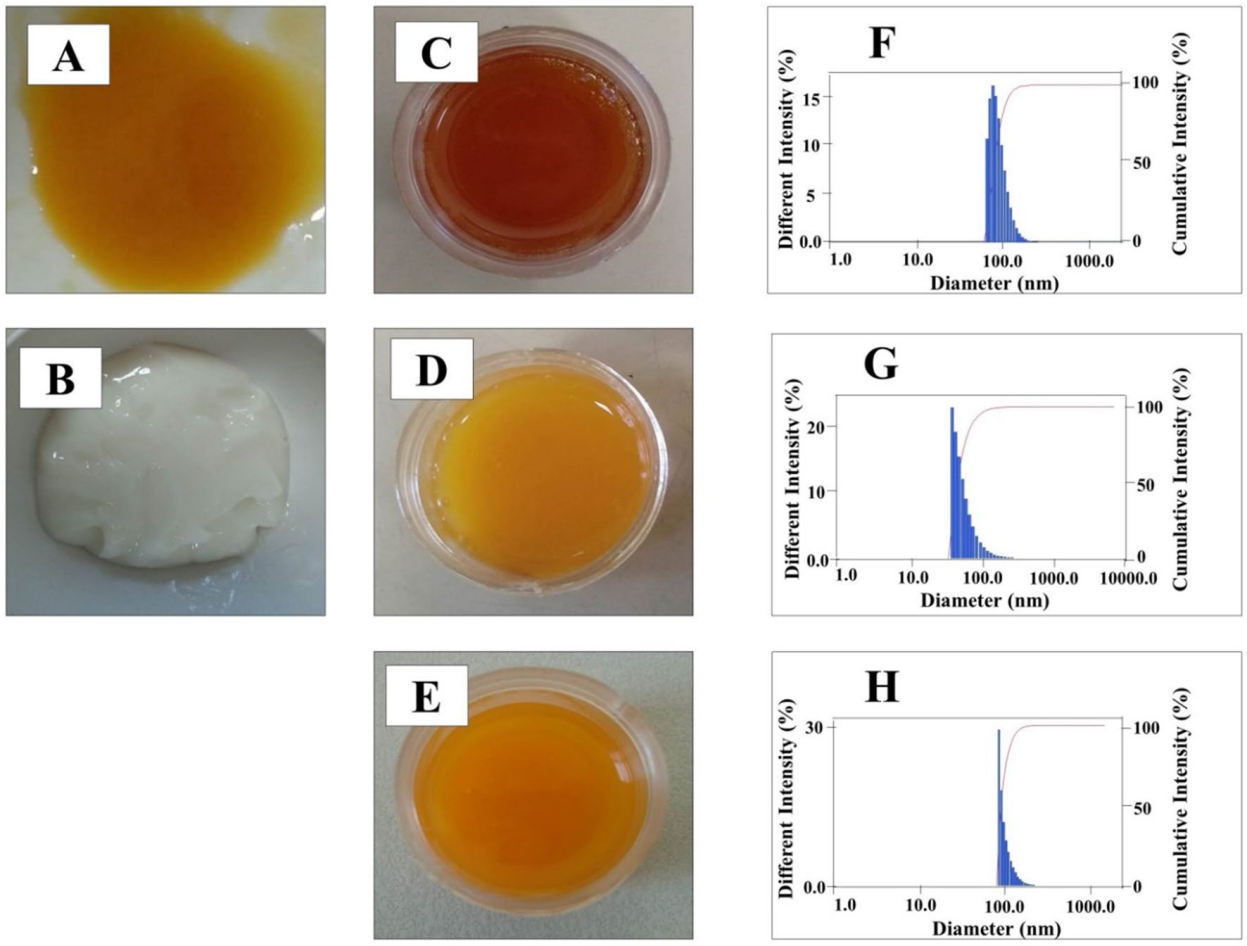
Visual appearance of protransfersomal CoQ10 (Protransf-CoQ10) (**A**), emulgel base (**B**), protransfesomal CoQ10 (Protransf-CoQ10) Emulgel (**C**), CoQ10 dissolved in oleic acid (CoQ10-Ole) Emulgel (**D**), and CoQ10 loaded in emulgel (CoQ10 Emulgel) (**E**). The Intensity distribution of particle of protransfesomal CoQ10 (Protransf-CoQ10) Emulgel (**F**), CoQ10 dissolved in oleic acid (CoQ10-Ole) Emulgel (**G**), and CoQ10 loaded in emulgel (CoQ10 Emulgel) (**H**).

The dispersion of Protransf-CoQ10 into the emulgel base (Fig. 2A, B) at a weight ratio of 2:1 produced Protransf-CoQ10 Emulgel whose color changes to brownish orange with a reduction in its pungent smell as shown in Fig. 2C. CoQ10 dissolved in oleic acid (CoQ10-Ole) was in the form of a bright orange odorless emulgel (Fig. 2D) whose character is identical to that of CoQ10 Emulgel except that it is more transparent due to no oleic acid being present in the formula (Fig. 2E). The darkening color of Protransf-CoQ10 emulgel probably due to large amount of L-α-Phosphatidylcholine content of which is dark yellow in color^30^ and easily oxidized when it is exposed to air in for lengthy periods^31,32^.

The particle size and polydispersity index value were further evaluated since they determine the ability of the vesicles to penetrate the deeper layers of the skin. The smaller the particle size of the vesicles, the easier the vesicles are to penetrate. In addition, the smaller the polydispersity index value, the more homogeneous the particle size of the vesicles^17^, thus ensuring that a larger number of vesicles penetrate the skin. From the results, it is evident that the entrapment efficiency value of the CoQ10 in Protransf-CoQ10 is comparatively high at 45.64 ± 7.52% with particle size of 201.5 ± 6.1 nm (by manual shaking method), polydispersity index value of 0.229 ± 0.047, and ζ-potential of − 11.26 ± 5.14 mV, as presented in Table 1. The manual shaking method of 5 min duration was reflective of the real situation in which protransfersomes change into transfersomes. The Protransf-CoQ10 Emulgel had the smallest particle size compared to both CoQ10-Ole Emulgel and CoQ10 Emulgel, which were 134.3 ± 4.8 nm < 146.9 ± 1.6 nm < 238.8 ± 3.1 nm, respectively, with intensity distribution of particle presented in Fig. 2F–H. The polydispersity index values for Protransf-CoQ10 Emulgel, CoQ10-Ole Emulgel, and CoQ10 Emulgel were 0.291 ± 0.020 < 0.298 ± 0.019 < 0.384 ± 0.010.

**Table 1:** Particle Size and polydispersity index of CoQ10 loaded in emulgel (CoQ10 Emulgel), CoQ10 dissolved in oleic acid (CoQ10-Ole) Emulgel, and protransfesomal CoQ10 (Protransf-CoQ10) Emulgel.

Formula	Particle size (nm)	Polydispersity index (PDI)
CoQ10 Emulgel	238.8 ± 3.1	0.384 ± 0.010
CoQ10-Ole Emulgel	146.9 ± 1.6	0.298 ± 0.019
Protransf-CoQ10 Emulgel	134.3 ± 4.8	0.291 ± 0.020

Each value represents the mean ± SD (n = 3).

**Figure 3:**
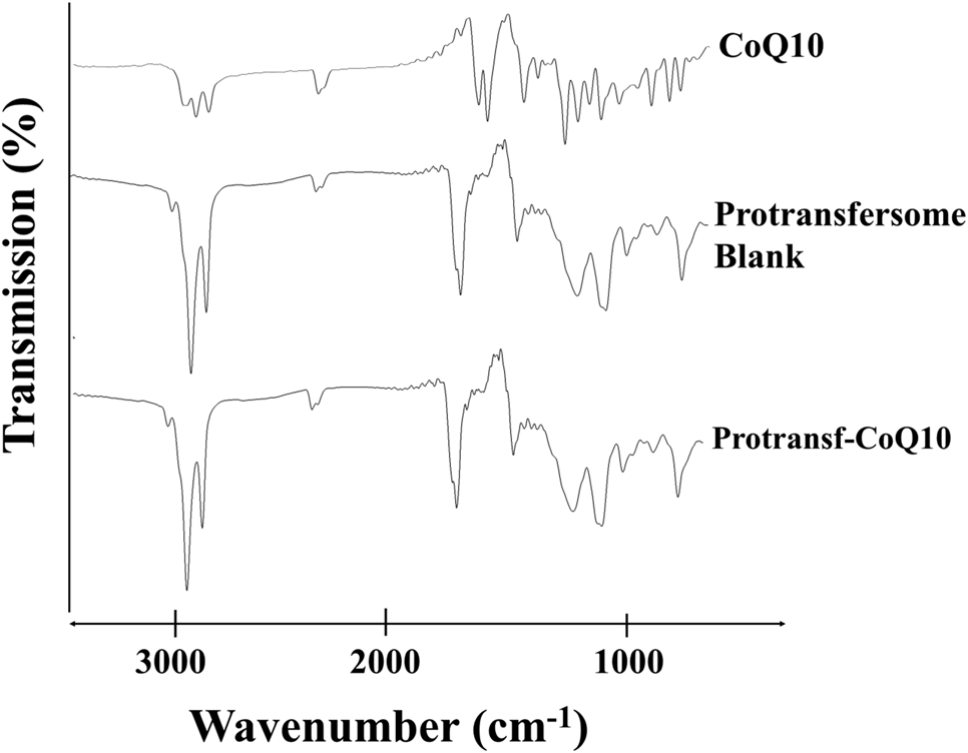
Fourier-transform infrared spectra of Coenzyme Q10 (CoQ10), Blank protransfersome, and protransfersome loaded CoQ10 (Protransf-CoQ10).

In order to evaluate any interaction between CoQ10 and protransfersomal matrix, a Fourier Transform Infra Red (FTIR) analysis was further observed. As presented in Fig. 3, there were no new absorption bands of functional groups or peak shifts observed for Protransf-CoQ10, which shows similar infrared spectroscopical profiles to Protransfersome blank, while no specific peaks of CoQ10 appear. This result indicates that CoQ10 successfully encapsulated protransfersome and no chemical interaction between the mixtures occurred^33–35^.

**Figure 4:**
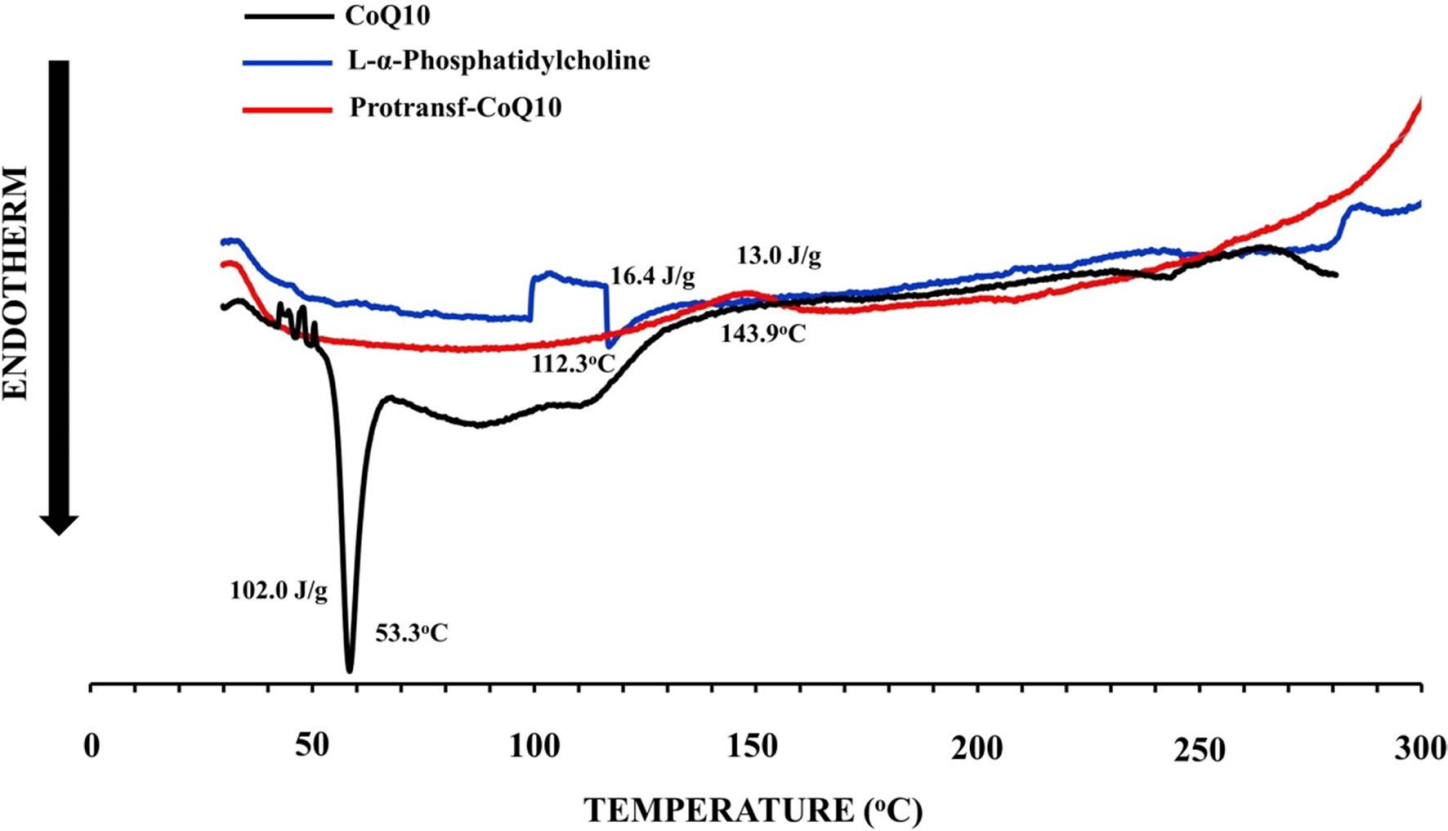
Differential thermal analysis of Coenzyme Q10 (CoQ10), L-α-Phosphatidylcholine as phospholipid component of protransfersome, and protransfersome loaded CoQ10 (Protransf-CoQ10).

Moreover, according to the result of differential thermal analysis, the CoQ10 encapsulation into protransfersome produced changes in the structure of cristallinity. CoQ10 and L-α-Phosphatidylcholine showed sharp endothermic peaks at 53.3 and 112.3 °C, respectively; however, protransfersomal CoQ10 showed weak endothermic peak at 143.9 °C indicating that less ordered crystalline structures were observed as presented in Fig. 4.

**Figure 5:**
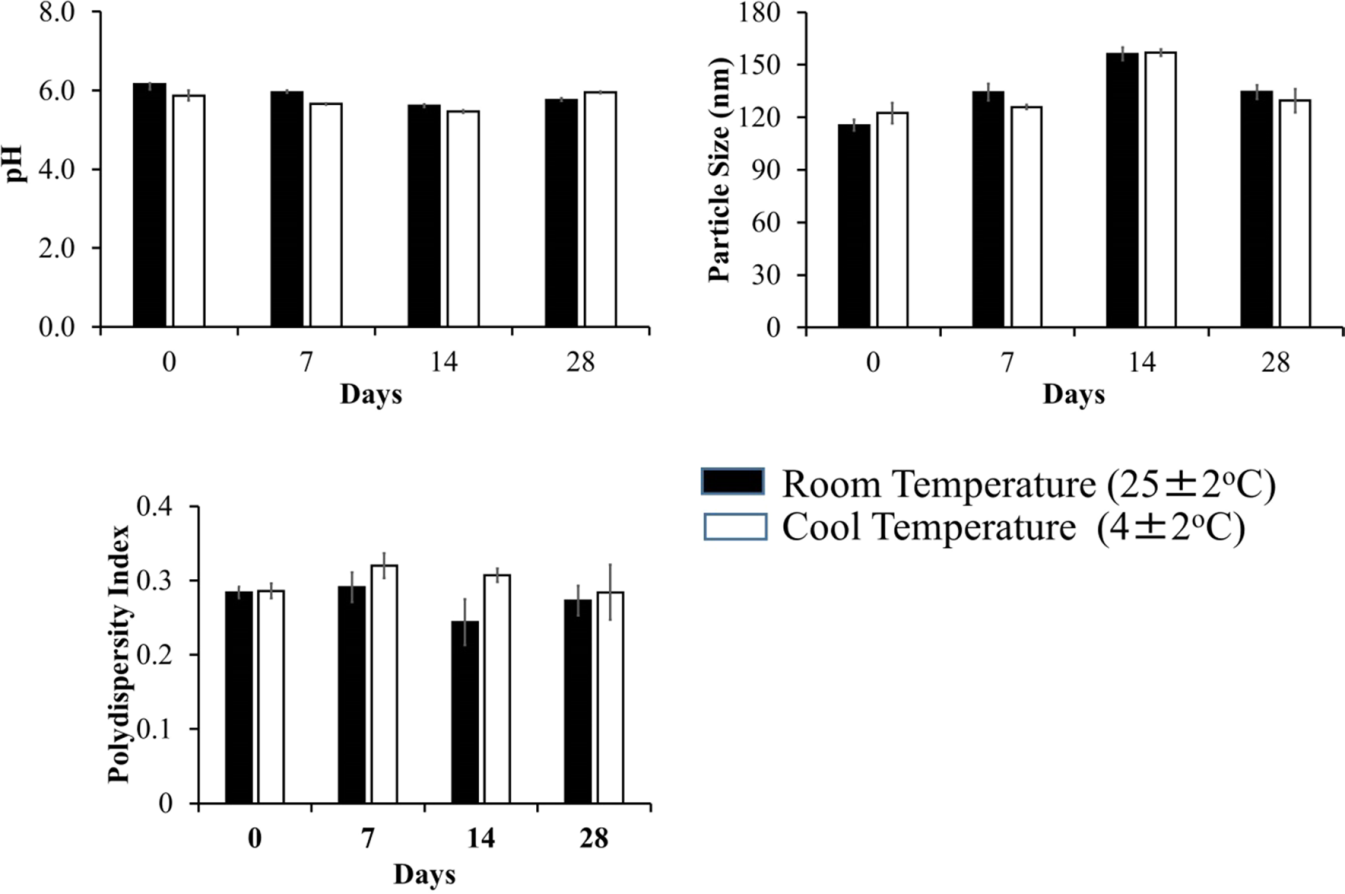
Evaluation of particle size, polydispersity index, and pH stability of protransfersomal CoQ10 loaded in emulgel (Protransf-CoQ10 Emulgel) during 28 days stored at room (25 ± 2 °C) and cool (4 ± 2 °C) temperatures.

A physical stability test was subsequently carried out to determine the physical resistance of the system when stored at different temperatures, namely; room temperature and a lower temperature for 28 days. During the study, the parameters of particle size, polydispersity index, and pH were observed. As seen from Fig. 5, the results showed that after a 28-day storage period, there were no significant differences in particle size or polydispersity index (*P* < 0.05). On the other hand, a significant difference was observed in the pH during the same period, although the pH value remained within the pH range of the skin. No significant difference existed in the particle size or particle size distribution of the preparation after 28 days of storage.

### In vivo anti-ageing activity of protransfersome-loaded CoQ10 emulgel

**Figure 6:**
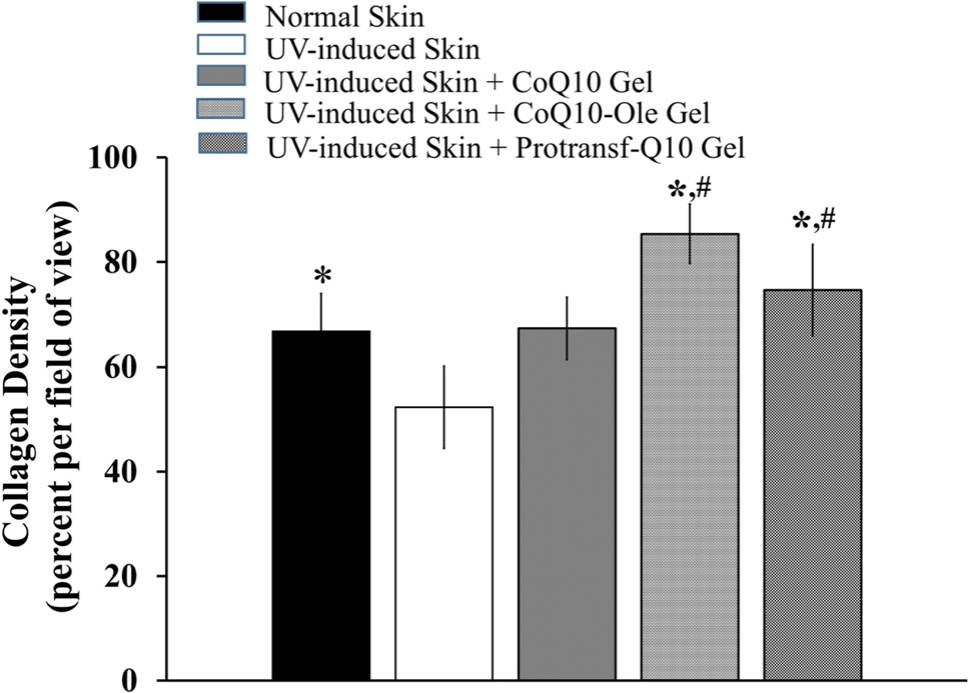
The collagen density of dermis layer of subject’s back skin without and with UV-induced photoageing after topically applied with saline (Normal skin and UV-induced skin), CoQ10-loaded Emulgel, CoQ10 dissolved in oleic acid (CoQ10-Ole) Emulgel, and protransfesomal CoQ10 (Protransf-CoQ10) Emulgel once every 2 days for 2 weeks. *P < 0.05 compared to UV-induced skin, #*P* < 0.05 compared to Normal Skin.

To evaluate the ability of protransfersomes to topically deliver CoQ10 and produce an effective anti-ageing activity, the Protransf-CoQ10 Emulgel was topically applied for 14 days to the back skin of UV-rays-induced subjects who were subsequently observed for skin histopathology. The control group subjects which received UV rays had the lowest collagen density of 52.30 ± 7.87%, indicating that UV rays damage the collagen in the skin dermis. The administration of both Protransf-CoQ10 Emulgel and CoQ10-Ole Emulgel significantly improved the collagen density of UV-ray radiated subjects’ skin as indicated in Fig. 6. However, there was no significant difference between these groups. The use of protransfersomes successfully delivered CoQ10 providing protection against skin damage and repaired that resulting from exposure to UV rays.

**Figure 7:**
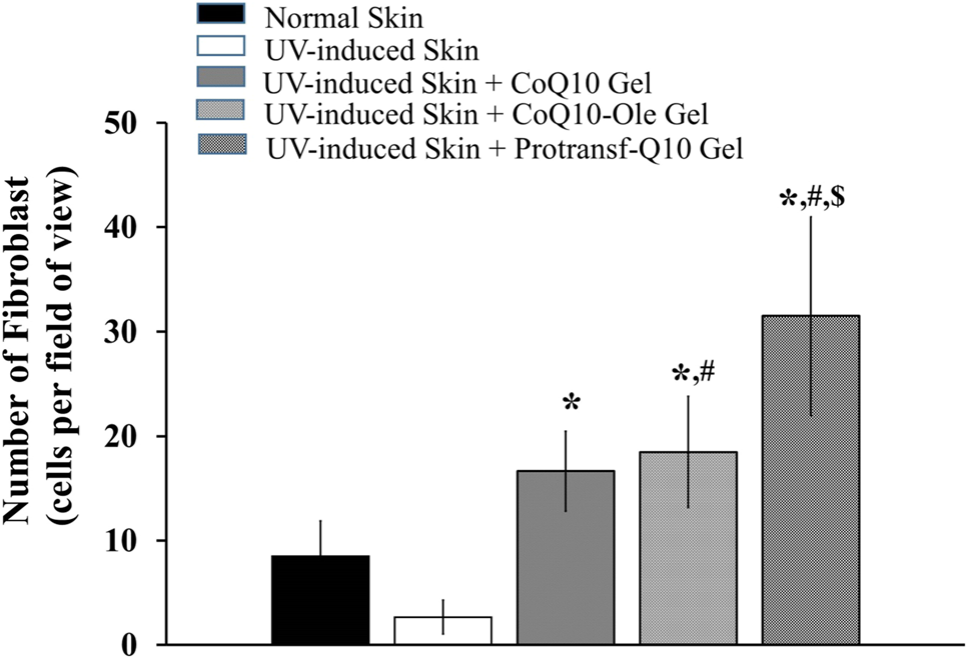
The number of fibroblasts of mice back skin without and with UV-induced photoageing after topically applied with saline (Normal skin and UV-induced skin), CoQ10-loaded Emulgel, CoQ10 dissolved in oleic acid (CoQ10-Ole) Emulgel, and protransfesomal CoQ10 (Protransf-CoQ10) Emulgel once every 2 days for 2 weeks. *P < 0.05 compared to UV-induced skin, #*P* < 0.05 compared to Normal Skin, $ *P* < 0.05 compared to CoQ10-Ole treated skin.

The anti-ageing activity test result was further analyzed by observing the number of fibroblast cells capable of producing collagen. Therefore, the higher the number of fibroblasts, the more collagen was formed. In this study, the assessed fibroblasts were young and light purple in appearance. The results showed that the CoQ10 Emulgel had a significantly different number of fibroblasts compared to the control group, with pro-CoQ10 Emulgel producing the highest number of fibroblasts, which was 31.50 ± 9.48 cells per field view, as indicated in Fig. 7. This shows that protransfersomes delivering CoQ10 successfully increase the number of fibroblasts.

### In vivo skin irritation test

**Figure 8:**
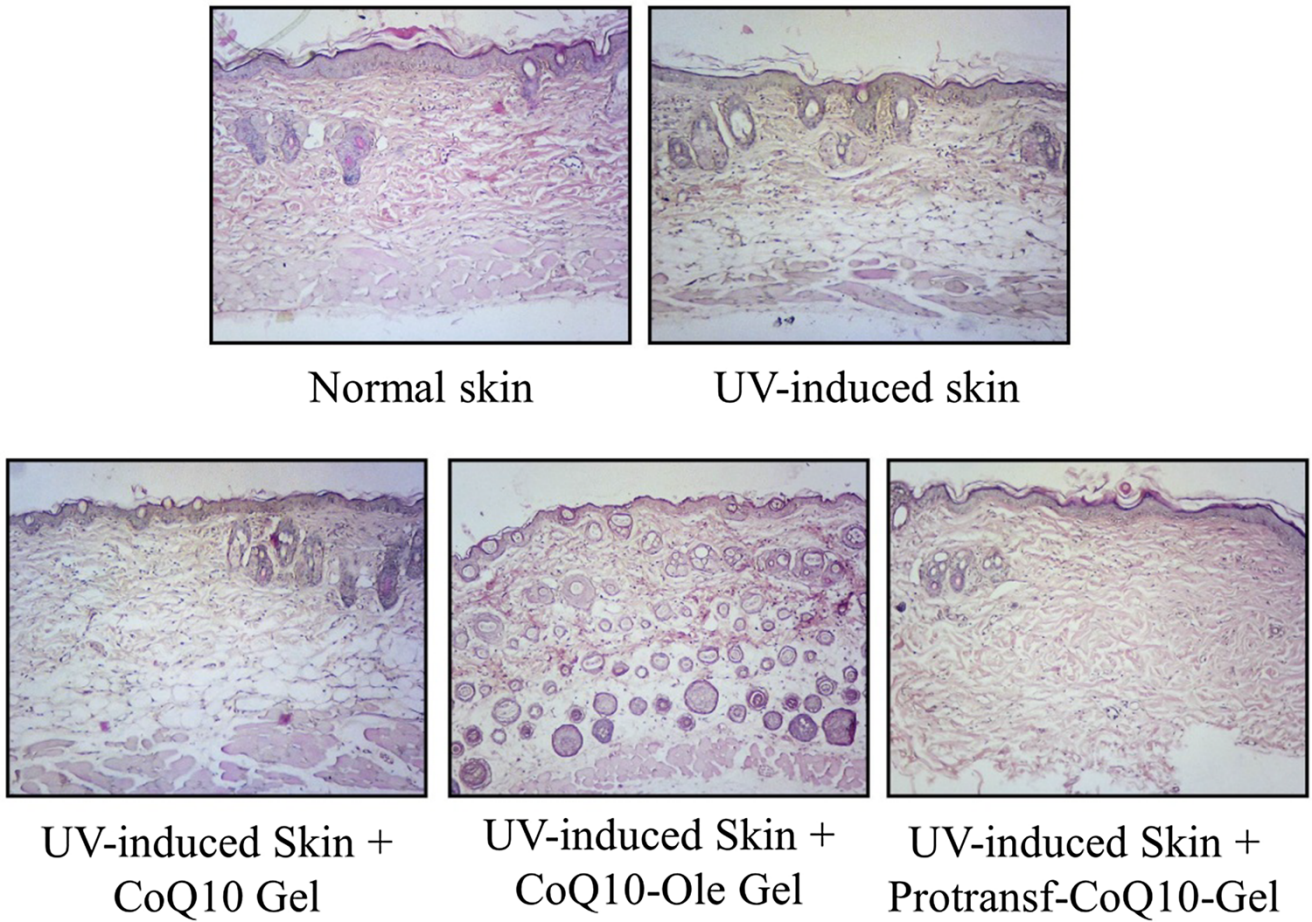
The histopathology of mice back skin stained with hematoxylin–eosin without and with UV-induced photoageing at 24 h after topically applied with saline (Normal skin and UV-induced skin), CoQ10-loaded Emulgel, CoQ10 dissolved in oleic acid (CoQ10-Ole) Emulgel, and protransfesomal CoQ10 (Protransf-CoQ10) Emulgel.

The safe use of Protransfersome-loaded emulgels in this study was also evaluated by conducting an in vivo irritation test. Epidermis liquefaction, subepidermis edema, collagen fiber swelling, inflammatory cells infiltration, dan appendages degeneration were observed for determining irritation in model’s skin. As presented in Fig. 8, there are differences in skin histopathology between normal and UV-induced skin. For further evaluation of severity level of skin irritation, scoring was then determined for each group.

**Figure 9:**
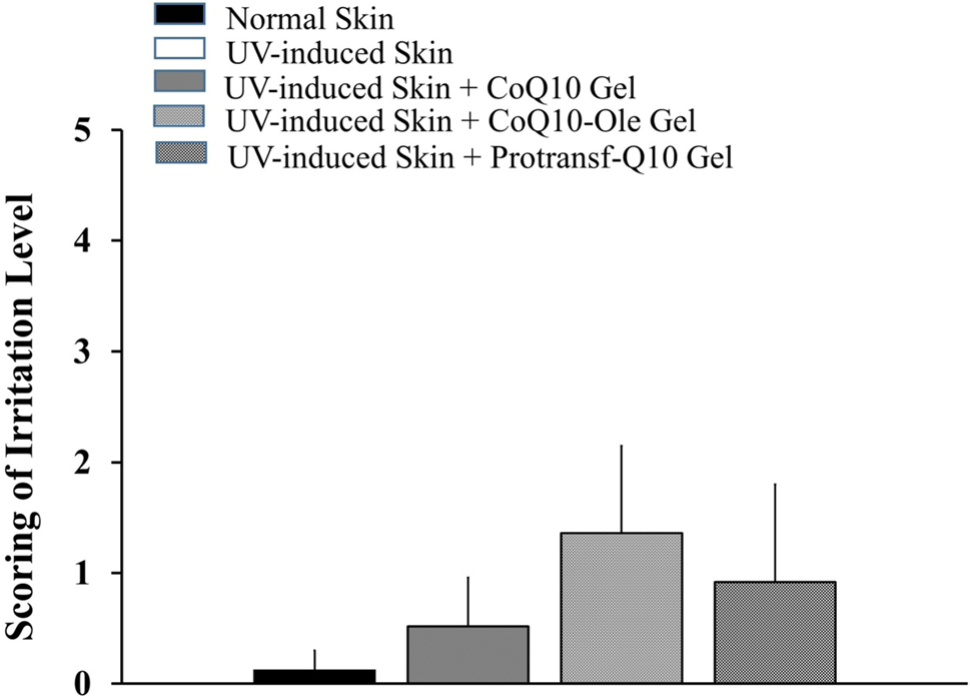
The scoring results of histopathology of mice back skin s without and with UV-induced photoageing at 24 h after topically applied with saline (Normal skin and UV-induced skin), CoQ10-loaded Emulgel, CoQ10 dissolved in oleic acid (CoQ10-Ole) Emulgel, and protransfesomal CoQ10 (Protransf-CoQ10) Emulgel.

The results of the histopathological scoring of the models’ back skin after 24 h of application showed that CoQ10 Emulgel had an irritation score of 0.52, while CoQ10-Ole Emulgel had one of 1.36, and Protransf-CoQ10 Emulgel one of 0.92 as presented in Fig. 9. This result shows that the Protransf-CoQ10 Emulgel does not irritate the skin, while the CoQ10-Ole Emulgel induced mild irritation due to the nature of oleic acid. According to the Kruskall Wallis statistical test results, there was no significant difference between these emulgel preparations.

## Discussion

In this study, the Protransfersomes and Protransfersomal emulgel preparations for CoQ10 delivery as the active cosmetic ingredient have the potential to inhibit premature ageing of the skin. The main purpose of protransfersome formulation is to significantly encapsulate CoQ10 in order to modify the physicochemical characteristics of CoQ10, rendering it more water dispersible and able to penetrate the skin since high lipophilic CoQ10 demonstrates low water solubility and poor skin penetration. However, the high content of oleic acid, which accounted for approximately 37% of the final weight of protransfersomal emulgel, would render it unacceptable for daily use as a skin cosmetic. Therefore, it was added to emulgel to increase its appropriateness for use. As far as the functional aspects of vesicles are concerned, the formation of transfersome due to hydration of protransfersome by water content in the emulgel base produces ultra-deformable vesicles which allow them to easily penetrate the skin. In addition, previous reports showed that the presence of a gelling agent would act as a steric hindrance which would be adsorbed onto the vesicle surface preventing fusion or aggregation, thus increasing physical stability during storage^36,37^. The addition of lipid vesicles to gel is beneficial for increasing vesicle stability, prolonging drug release, improving dermal permeability, and enhancing drug deposition in the skin^38^.

Protransfersomes have been developed as the nanometer-sized carrier form of transfersome provesicles and have a higher phospholipid content compared to transfersomes. This enables the protransfersome system to demonstrate greater entrapment efficiency due to a higher number of vesicles formed that are subsequently available for encapsulating drugs, thus providing high stability when compared to the transfersome system^24^. Protransfersomes are able to carry active ingredients through the skin pores into the deeper layer. The protransfersome system analyzed in this study has positive characteristics including nanometer size, and thick consistency resulting from its large phospholipid content. When the protransfersome is observed using a light microscope, a palisade lamellar structure appears in the form of liquid crystals. This is due to differences in the degree of hydration of surfactants and phospholipid molecules triggered by solvent limitations. The protransfersome forms as a mixture of flat liquid crystals resembling palisade and vesicular lamellae linked together^39^. The percentage of entrapment efficiency (EE%) of the protransfersome system is a parameter used to predict the stability of the dispersion^40^ describing the amount of drug present in the vesicle^41^. In this study, the EE% value was comparatively large because it corresponded to the phospholipid content in the formula^42^ and the tendency of CoQ10 to be retained in the phospholipid membrane due to its lipophilic properties^39^.

To improve acceptability, the protransfersome was formulated as an emulgel preparation incorporating the use of an emulgel as the gel base. In this study, three types of emulgels were developed and evaluated for their anti-ageing and irritability activity, namely; Protransf-CoQ10 emulgel, emulgel loaded CoQ10 which was previously dissolved in oleic acid (CoQ10-Ole emulgel) and CoQ10 dispersed in an emulgel base (CoQ10 emulgel).

During the homogenization method for preparing necessary samples the particle size test involves manual shaking which is considered to closely replicate real-life conditions. The particle size of the emulgel loaded Co-Q10 remained in the nanometer range, indicating that adding emulgel base to the particle size of Protransf-CoQ10 had no effect. The particle size of Protransf-CoQ10 Emulgel is smaller than that of Protransf-CoQ10 itself. This indicates that the particles have turned into transfersome vesicles because they have been partially hydrated by the presence of water in the emulgel base. The decreased vesicle size of protransfersomal CoQ10 after dispersion into the emulgel base is probably due to the shearing stress that occurs during the incorporation of Protransf-CoQ10 into hydrated Carbopol-based emulgel. This causes the small vesicles formed and the emulgel matrix to be adsorbed onto the vesicle surface, preventing vesicle fusion or aggregation^36,37^, while spontaneous hydration of protransfersome produces larger vesicles than those resulting from dispersion into emulgel. When compared to the particle sizes of CoQ10-Ole Emulgel and Co-Q10 Emulgel, those of all three emulgels-loaded CoQ10s can be measured in nanometers. The order of particle size from the smallest to the largest is Protransf-CoQ10 Emulgel < CoQ10-Ole Emulgel < Co-Q10 Emulgel. Co-Q10 Emulgel is the largest in size because CoQ10 is only dispersed in the emulgel base, while Protransf-CoQ10 Emulgel and CoQ10-Ole Emulgel had similar particle size and PDI probably due to CoQ10 solubility in Oleic Acid for both formulas^43^. From the results of the polydispersity index, it is evident that all particles have a uniform size distribution. This indicates that the preparation will be stable during storage because it reduces the tendency for particle aggregation which causes the system to become unstable.

A test was carried out to determine the physical stability of Protransf-CoQ10 emulgel when stored at different temperatures, namely; room temperature and colder temperatures for 28 days and whether differences in particle size, polydispersity index, and pH existed. There was no significant difference in particle size, polydispersity index, and pH of Protransf-CoQ10 emulgel during the study period.

The results of the anti-ageing activity of CoQ10 loaded in emulgel and evaluated for skin collagen density confirmed CoQ10-Ole Emulgel as having the highest percentage of collagen density, followed by Protransf-CoQ10 Emulgel. However, no significant difference existed between these groups (*P* > 0.05). These two groups demonstrated significant improvement in collagen density compared with the control group whose subjects had been exposed to UV and who recorded the lowest density value. This is probably due to soluble CoQ10 in Oleic Acid loaded into emulgel had been easily released from emulgel than that of Protransf-CoQ10 Emulgel, which the formation of vesicle during hydration results in semipermeable bilayer membrane as water diffusion-limiting barriers for CoQ10 release. The low collagen density has been known caused by imbalance between collagen synthesis by fibroblasts and collagen degradation of UV irradiation, while collagen synthesis is proportionally relate to fibroblasts resident^44^. Moreover, collagen synthesis by fibroblast will actively occur on the 4th day of 21 days^45^. The faster CoQ10 release from CoQ10 Ole Emulgel will stimulates fibroblast proliferation which increase expression of collagen matrix^46^, while the late CoQ10 release from Protransf-CoQ10 Emulgel will result in delayed effects on fibroblast-stimulated collagen synthesis.

On the other hand, the Co-Q10 Emulgel-treated group had similar collagen density to that of normal mice, indicating that UV light damages collagen in the skin dermis. It has been known that UV-irradiation damage dermal collagen and elastin fibers^47^, while CoQ10 increased the collagen content through decrease of MMP‐1 protein level in mice exposed with UV-B^48^. CoQ10 also promotes the fibroblast proliferation^49^. However, it seems that the fibroblast stimulation process to produce collagen matrix between normal and CoQ10-treated groups is different. This situation differed from that of the group treated with CoQ10 in the emulgels. From these results, it can be concluded that CoQ10 provides protection against the ageing effects of UV rays.

The anti-ageing activity test was further evaluated for the number of fibroblasts in the skin tissues. Fibroblasts are cells capable of producing collagen. In this case, the assessed fibroblasts were young and light purple in color. The higher the number of fibroblasts, the more collagen was formed. The results showed that the CoQ10 emulgels had a significantly different number of fibroblasts compared to the control group, with the Protransf-CoQ10 Emulgel having the highest number, which was 31.50 ± 9.48% per field view. This indicates that CoQ10 is able to increase the number of fibroblasts.

The safety of these anti-ageing emulgels was further evaluated by an irritancy test. The results indicated that the Protransf-CoQ10 Emulgel produced no signs of irritation in the skin tissues observed, while the CoQ10-Ole Emulgel induced mild skin irritation due to the nature of oleic acid.

Protransf-CoQ10 Emulgel has potential as an anti-aging product. However, information is lacking about both the drug release profile and its dermal penetrability which supports the theory that protransfersome and its incorporation into emulgel could prove a useful model for developing skin anti-aging cosmetics. Moreover, both the ability of protransfersome and protransfersomal emulgel to maintain drug stability and the physicochemical properties of the forms of skin dosage need to be evaluated for drug levels during study periods in line with ICH guidelines. Therefore, the product development involved could be comprehensively analyzed.

## Conclusions

The results of this study indicate that emulgel-loaded protransfersomes, employed as delivery carriers of CoQ10, possess positive physical properties, thereby increasing anti-ageing activity with a low skin irritancy score. Proposing the incorporation of protransfersomal emulgel into cosmetics requires further studies especially on the acceptability test in humans and stability tests for longer storage times. From the results of this study, although the primary nature of CoQ10 severely limits its skin delivery, protransfersome provides potential benefits when used as a delivery system for active cosmetic ingredients within skin ageing therapy.

## Methods

### Materials

In this study Coenzym Q10 (CoQ10) was obtained from Kangcare Bioindustry Co. Ltd. (Nanjing, China). L-α-Phosphatidylcholine is a product of Sigma-Aldrich (Buchs, Switzerland). Tween 80 and Span 80 were both purchased from Enviro Prima Co. Ltd. (Tangerang, Indonesia). The oleic acid used in this study was acquired from Brataco Co. Ltd. (Surabaya, Indonesia). All other reagents were of the available pharmaceutical and analytical grades.

### Preparation of CoQ10-loaded protransfersome (Protransf-CoQ10)

The protransfersome was composed of L-α-Phosphatidylcholine, Oleic Acid, and Tween 80 as shown in Table 2 and prepared with modifications by the method previously reported by Gupta (2012)^16^. Initially, CoQ10 was stirred until completely dissolved in a mixture of oleic acid and Tween 80. Finally, L-α-Phosphatidylcholine was added and stirred until dissolved to produce Protransf-CoQ10.

**Table 2:** Formulation of CoQ and protransfersomal CoQ10-loaded emulgels.

Component	Amount in formula (%)		
	Protransf-CoQ10 Emulgel	CoQ10-Ole Emulgel	CoQ10 Emulgel
Coenzyme Q10	1.0	1.0	1.0
L-α-Phosphatidylcholine	24.9	–	–
Oleic acid	37.2	37.2	–
Tween 80	4.3	4.3	–
Emulgel base	Up to 100.0	Up to 100.0	Up to 100.0

### Preparation of emulgel containing CoQ10-loaded protransfersome (Protransf-CoQ10 Emulgel)

A CoQ10-loaded protransfersome emulgel was prepared by adding the Protransf-CoQ10 to the emulgel base with a final CoQ10 content of 1%. The emulgel base was produced using Carbopol 940 added to a combination of Tween 80 and Span 80 (1:1) to form a homogenous emulgel base with the addition of Triethylamine (TEA) to adjust the pH to 6.0 ± 0.2. Protransf-CoQ10, CoQ10 solution in oleic acid, and CoQ10 powder were subsequently added to this emulgel base and mixed homogenously to produce Protransf-CoQ10 emulgel, CoQ10-Ole emulgel, and CoQ10 emulgel, respectively.

### Evaluation of physical characteristics

The evaluation of physical characteristics includes particle size, polydispersity index, ζ-potential, microscopic observation, entrapment efficiency, and physical stability during storage.

The dispersion of Protransf-CoQ10 into an emulgel base at a weight ratio of 2:1 produced Protransf-CoQ10 emulgel whose color changes to brownish orange and the reduction on its pungent odor. Meanwhile, the CoQ10 dissolved in oleic acid (CoQ10-Ole) emulgel had an odorless, jelly-like consistency and was bright orange in color. These characteristics were identical to those of CoQ10 emulgel, although the latter had a more transparent appearance due to the absence of oleic acid from the formula.

Evaluation of particle size and ζ-potential were respectively carried out using a Delsa™ Nano Submicron Particle Size Analyzer (California, USA) and light scattering and electron scattering methods. Approximately 50 mg of CoQ10-loaded protransfersome and emulgels were resuspended in 5 mL of 0.9% NaCl. The samples were then prepared using the manual shaking method for 5 min^24^. The suspension was further diluted by pipetting 150 μL of sample and added with 2 mL of deionized water (Otsuka Indonesia, Lawang, Indonesia) for sample measurement.

The Protransf-CoQ10 was observed microscopically to evaluate its transformation ability in relation to transfersome vesicles by placing a small amount of sample on a glass slide and covering it with a cover glass. A drop of 0.9% NaCl saline solution was added to the other side of the cover slip’s cavity^50^. The evaluation was conducted using an optical microscope before, during, and after addition of 0.9% NaCl at 400 × magnification.

The EE% was measured for CoQ10 loaded in protransfersome by means of UV–Vis spectrophotometry^17^. Approximately 100 mg of Protransf-CoQ10 was weighed, and then hydrated with 2 mL phosphate buffered saline (PBS) pH 7.4 and sonicated for 30 min until homogeneous. The suspension formed was then centrifuged at 3000 rpm for 30 min to obtain supernatant and sediment in a 10 mL glass tube. The sample was prepared by taking 1.5 mL of supernatant and then dissolved in 2 mL methanol, added to 2 mL PBS pH 7.4 and, finally, sonicated for 15 min. The sediment was dissolved in 1.5 mL methanol, added to 2 mL of PBS and sonicated for 15 min. The absorbance of each sample was measured by UV–Vis spectrophotometry at a wavelength of 275 nm. The EE (%) of CoQ10-loaded in protransfersome was calculated by means of the following equation:

1 $${\text{EE}}\, (\%)= \frac{CoQ10\;levels\;in\;supernatant}{{CoQ10\;levels\;in\;supernatant + CoQ10\;levels\;in\;sediment}} \times 100\%$$

In order to evaluate whether any chemical or physical changes occurred in samples, spectroscopical and thermal analysis were further investigated. The spectroscopical analysis was evaluated using a Fourier Transformed Infra-Red analysis by using Spectrophotometer ECO ATRS Bruker Alpha II (Germany). About 1 mg sample was analyzed at wavenumbers of 450–4000 cm^−1^. While, the thermal analysis was evaluated using *Differential Thermal Analysis* (DTA) instrument (Mettler Toledo FP 85, Switzerland). About 3–5 mg samples was put into crucible sample pan. The sample was then subsequently heated from 30 to 300 °C at a heating rate of 10 °C per minutes.

Moreover, a stability test of the Protransf-CoQ10 emulgel was carried out by storing the samples at in the dark at room temperature (24 ± 2 °C) and, subsequently, a cold temperature (4 ± 2 °C) for 28 days^51–53^. The emulgel was evaluated for physical characteristics, i.e., pH and particle size, on the 28th day after preparation.

### In vivo study of anti-ageing in UV-rays ageing induced mice

The in vivo anti-ageing activity was evaluated using Balb/c mice (*Mus musculus*) within the terms of a study protocol approved by The Ethics Commission of Faculty of Veterinary Medicine, Universitas Airlangga (Certificate number 2.KE.016.02.2020 dated February 4, 2020). All methods were performed in accordance with ARRIVE guidelines and relevant regulations^54^. Within this research, two types of study involving the uses of experimental models were evaluated, firstly, anti-ageing activity as indicated by collagen density and number of fibroblasts, and, secondly, a safety test incorporating irritancy scoring of skin tissue. The effect of the Protransf-CoQ10 emulgel was compared with those of CoQ10-Ole and CoQ10 emugels. Each group comprises of 4 mice as the study model. Prior to the study, the hair on the models’ backs was trimmed with mechanical hair clippers, ensuring that their skin was not injured during this process. Each model was housed in a separate cage to prevent their touching the part to be smeared with the sample.

### Anti-ageing activity test

The anti-ageing activity test was evaluated to establish the parameters of collagen fiber density and the number of fibroblasts. The study was carried out by applying 200 mg of the emulgels twice a day every day to a 4 cm^2^ area of previously shaved skin on the models’ backs. The sample was applied 20 min before UV irradiation, in order to provide time for absorption into the skin, and four hours after irradiation which is the point at which the formation of Reactive Oxygen Species commences. An 80 mJ/cm^2^ dose of UV light was administered at an irradiation distance of 15 cm for 21 min. UV irradiation was carried out once every 2 days, namely; on days 1, 3, 5, 7, 9, 11, and 13, with the models subsequently being left for 24 h on completion of the irradiation process to overcome the effects of acute irradiation^55^. Sample application was also conducted on days when the models were not exposed to UV irradiation. After 14 days, the models were sacrificed by dislocation with the skin tissues being subsequently excised to produce a tissue section using a microtome. To evaluate the collagen density, the tissue section was stained with Masson trichrome staining, while for the observation of fibroblasts, the skin tissue section was stained with Hematoxylin–Eosin Staining. The tissue section was then observed with a light microscope (Olympus CX 31 Camera DP 22) using Cellsen Standard Software. Collagen density was measured by histochemical scoring, while the number of fibroblasts was calculated by digital analysis using Adobe Photoshop and Image J software. Density measurement involved measuring the area of collagen coir and comparing it with the field of view. The denser the coir collagen, the higher the density value, and vice versa. Calculation of the density value was completed by means of calculating the area of the field of view and the black colored area using Image J software calibrated fin advance or each degree of magnification. The comparison of the black stained area with the field of view produced the density value.

### In vivo skin irritancy evaluation of proransfersome loaded CoQ10 in emulgels

In order to observe the irritant effects of CoQ10-loaded in proransfersome and emulgels, histopathological changes in the skin tissues of each model after a 24-h period of exposure were observed. Firstly, the back hair of the models had been shaved. Approximately 200 mg of the sample was then applied to a 2 × 2 cm^2^ area of skin on their backs. Twenty-four hours after application, the models were sacrificed by dislocation. Skin was excised with a microtome before being immersed in a formalin solution and stained with hematoxylin–eosin. The preparations were observed with a light microscope to assess the degree of skin irritation by means of histopathological scoring. Histological change data is semi-qualitative and features five variables, namely; epidermis liquefaction, subepidermal edema, collagen fiber swelling, inflammatory cell infiltration, and degeneration of the appendages in hair vesicles. The scoring method comprised a score of 0 = normal skin, 1 = mild irritation, 2 = moderate irritation, and 3 = severe irritation^56^. The data from each sample consisted of the mean value of the variable score for each of the five different fields of view at 100 × and 400 × magnification. All examinations involved the use of an ordinary light microscope (Nikon H600L, equipped with a 300 megapixel DS Fi2 digital camera and Nikkon Image System image processing software).

### Statistical analysis

The data in this study consisted of three replicates. In order to test the significance of differences in the data relating to Protransf-CoQ10 emulgel, CoQ10-Ole emulgel, and CoQ10 emulgel, a statistical analysis was performed using the one-way variant analysis (ANOVA) method. After the normality and homogeneity of the data had been tested, a Post Hoc Tukey HSD test was administered. If the *P value* < 0.05, then a significant difference between the results of the tests performed existed. However, if the data was not normally distributed and homogeneous, the data would be analyzed using non-parametric statistics by means of the Kruskall Wallis method and, subsequently, a Post Hoc Mann Whitney U test. If the *P* value < 0.05; then a significant difference existed.

### Ethical conduct of research statement

The animal study procedures were performed in accordance with the ethical clearance issued by The Ethics Commission of Faculty of Veterinary Medicine, Universitas Airlangga (Certificate number 2.KE.016.02.2020 dated February 4, 2020).
